# Communicating uncertainty in epidemic models

**DOI:** 10.1016/j.epidem.2021.100520

**Published:** 2021-12

**Authors:** Ruth McCabe, Mara D. Kont, Nora Schmit, Charles Whittaker, Alessandra Løchen, Patrick G.T. Walker, Azra C. Ghani, Neil M. Ferguson, Peter J. White, Christl A. Donnelly, Oliver J. Watson

**Affiliations:** a Department of Statistics, University of Oxford, 24–29 St Giles’, Oxford OX1 3LB, UK; b NIHR Health Protection Research Unit in Emerging and Zoonotic Diseases, The Ronald Ross Building, University of Liverpool, 8 West Derby Street, Liverpool L69 7BE, UK; c MRC Centre for Global Infectious Disease Analysis & WHO Collaborating Centre for Infectious Disease Modelling, Abdul Latif Jameel Institute for Disease and Emergency Analytics, Imperial College London, St Mary’s Campus, Norfolk Place, W2 1PG London, UK; d NIHR Health Research Protection Unit in Modelling and Health Economics, Imperial College London, St Mary’s Campus, Norfolk Place, London W2 1PG, UK; e Modelling and Economics Unit, National Infection Service, Public Health England, London, UK

**Keywords:** Transmission modelling, COVID-19, Communicating uncertainty, Decision-making, Data visualisation

## Abstract

While mathematical models of disease transmission are widely used to inform public health decision-makers globally, the uncertainty inherent in results are often poorly communicated. We outline some potential sources of uncertainty in epidemic models, present traditional methods used to illustrate uncertainty and discuss alternative presentation formats used by modelling groups throughout the COVID-19 pandemic. Then, by drawing on the experience of our own recent modelling, we seek to contribute to the ongoing discussion of how to improve upon traditional methods used to visualise uncertainty by providing a suggestion of how this can be presented in a clear and simple manner.

## Highlights

• Clearly communicating uncertainty to decision-makers is vital, but uncertainty is often poorly reflected in visualisations.
• Traditional summary statistics often hide the nuances of individual trajectories and important features of the epidemic.
• We present individual trajectories of ICU demand alongside individual metrics of magnitudes, peak timings and ICU capacity.
• To engage audiences with uncertainty in epidemic forecasts, we use colour to link each epidemic trajectory to key metrics.

## 1 Communicating uncertainty in epidemic models

During a public health crisis, such as an outbreak of a novel pathogen, decision-makers are often faced with making difficult and rapid decisions in the face of uncertainty (Craig, 2019). Mathematical models of disease transmission are one piece of evidence widely used to inform public health decision-makers globally, but, like all models, their outputs are uncertain (Zelner et al., 2021; Shea et al., 2020). Communicating this uncertainty during the COVID-19 pandemic in a clear and understandable manner to decision-makers is vital, but uncertainty has been often poorly reflected in the visualisations presented (Juul et al., 2021). The most important considerations when deciding on a data visualisation is knowing who the audiences are and ensuring that key messages can be easily and quickly absorbed (Craig, 2019). The pandemic has produced numerous examples of where statistics have been misunderstood and efforts to inform have ultimately confused audiences (Cheshire, 2020). One lesson that must be learnt from the pandemic is more effective ways to communicate quantitative findings and their uncertainty. Drawing on the experience of our own recent COVID-19 modelling, we seek to contribute to the ongoing discussion of how to improve upon traditional methods used to visualise uncertainty by providing suggestions of how this can be presented in a clear and simple manner.

The transmission of infections through a population is intrinsically complex and not directly observable. Transmission is often observed indirectly through recording cases of illness or death, or testing individuals for antibodies indicating history of previous infection. Mathematical models represent these processes by classifying the population into distinct states of infection and disease progression, with transitions between states governed by various epidemiological parameters. There are multiple parameter estimates and each is subject to uncertainty, so many sets of plausible combinations of parameter values exist. This uncertainty can be explored by producing multiple realisations of the model using different parameter sets. Uncertainty can also be introduced into simulations via the use of stochastic models, which, unlike deterministic models, incorporate the effects of random chance and are inherently ‘noisy’. The choice of model structure and parameterisation depends on the questions under consideration and often on the stage of the epidemic. For example, stochastic models were commonly deployed at the beginning of the COVID-19 pandemic, at which point there was limited understanding of the transmission dynamics of SARS-CoV-2 (Kucharski et al., 2020; Davies et al., 2020).

**Fig. 1:**
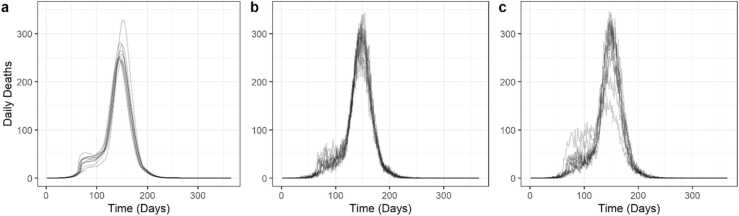
Uncertainty in epidemic models arising from different sources. Lines correspond to ten individual simulations of daily deaths over a one-year period using (a) a deterministic model with different sets of parameters per simulation; (b) a stochastic model with fixed parameters across simulations; and (c) a stochastic model with different sets of parameters per simulation.

Fig. 1 presents an illustration of ten simulation trajectories of daily deaths under three adaptations of a previously published model (Walker et al., 2020) in which uncertainty arises from different sources. Fig. 1a displays output from a deterministic model in which the uncertainty is driven by varying model parameters, whereas Fig. 1b presents the results of a stochastic model in which the parameters are held constant for each realisation. Finally, Fig. 1c presents a stochastic model in which model parameters are also varied. The combination of two sources of uncertainty lead to much greater variation in trajectories than that which is observed under the models with a single source of uncertainty. Regardless of the source and magnitude of the uncertainty in epidemic models, it is critical that uncertainty is always considered carefully in the presentation of any results. Additionally, depending on the audience and hypotheses under investigation, it could also aid the interpretation of results if the uncertainties in model inputs are also presented alongside model outputs.

**Fig. 2:**
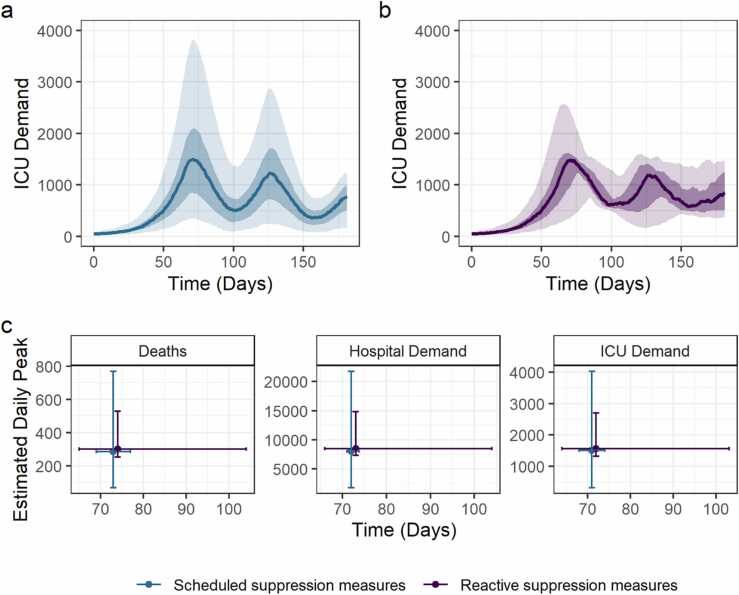
Traditional presentation of epidemic projections under two suppression strategies. The median (line) with 50% credible intervals (dark shading) and 95% credible intervals (light shading) of 100 simulations of intensive care unit (ICU) demand from COVID-19 patients is shown for strategies in which suppression measures are (a) scheduled at a fixed time point and (b) triggered reactively. The estimated median daily peak in deaths, hospital demand and ICU demand and the estimated time of each peak with 95% quantile ranges are shown under the two suppression scenarios in (c).

Results of epidemic models are most frequently summarised using simple summary statistics, such as the median and interquartile range. Drawing on our recent COVID-19 modelling work investigating intensive care unit (ICU) spare capacity in Europe (McCabe et al., 2021), we present an example of such an illustration in Fig. 2. Data are simulated daily hospital and ICU demand from COVID-19 patients and COVID-19 deaths using a stochastic age-dependent susceptible-exposed-infected-recovered (SEIR) model of SARS-CoV-2 transmission. The model was fit to COVID-19 deaths and ICU demand, with 100 simulation trajectories presented. Simulation realisations were sampled from the posterior parameter space from model fitting and used to provide scenario projections under two hypothetical intervention policies: 1) suppression measures are introduced at scheduled points in time and 2) suppression measures are triggered based on the number of COVID-19 patients in ICUs. Trajectories are summarised using the median with 50% and 95% credible intervals (calculated as 25th and 75th and 2.5th and 97.5th centiles, respectively) at each time point of the simulation. Additionally, we have provided two metrics of importance to decision-makers: the timing and the magnitude of peak ICU bed demand per simulation, presented as point estimates and 95% credible intervals. The median trajectories are very similar in both scenarios (Figs. 2a and 2b; Supplementary Figure 1), as are the point estimates of the magnitude and timing of each peak (Fig. 2c). However, under the “scheduled” strategy there is greater uncertainty in the magnitude than the timing of the first peak whereas, the alternative “reactive” strategy has an additional layer of uncertainty, with there being heterogeneity in both the magnitude and timing of peaks (Fig. 2c). This allows decision-makers at a national-level to consider the possible burden different policies would place on the healthcare system, while also providing information to decision-makers at a hospital-level about the potential level of additional resources required to meet the projected surge in demand, conditional on the policy choice.

**Fig. 3:**
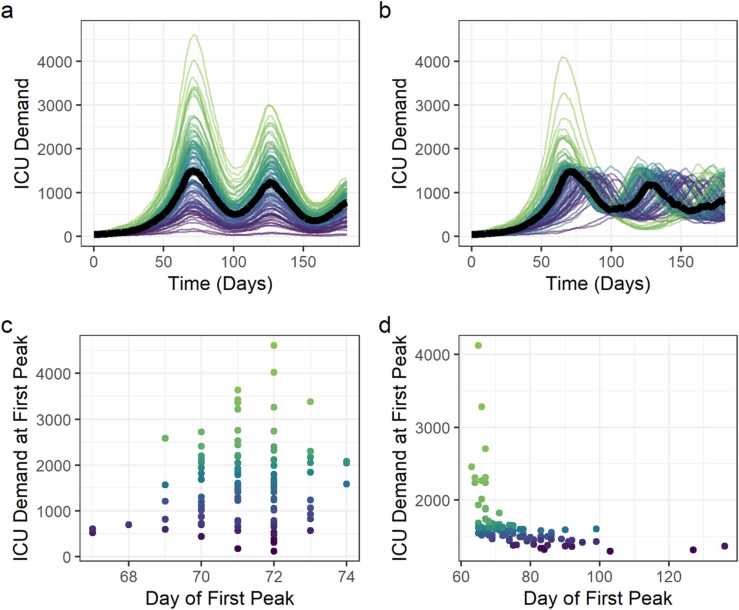
Communicating uncertainty in epidemic projections of ICU demand under two suppression strategies. Individual simulations of intensive care unit (ICU) demand from COVID-19 patients is shown for strategies in which suppression measures are (a) scheduled at a fixed time point and (b) triggered reactively, with corresponding relationships between the magnitude and timing of peak ICU demand for each realisation shown in (c) and (d), respectively. In (a) and (b) the median trajectory is shown with a black line. The colour of each simulation realisation depicts the ranking of ICU demand at first peak [high in green to low in purple] and allows for the metrics in (c) and (d) to be more easily linked to the trajectories in (a) and (b) respectively.

However, such aggregation often hides the nuances of individual trajectories as well as important features of the epidemic. For example, the typical peak number of infections across the set of individual trajectories may be drastically underestimated if results for multiple different, asynchronous epidemic trajectories are crudely summarised. We have encountered such issues in our work (McCabe et al., 2021) (Fig. 3) and hypothesise that this is also true of other research groups who have chosen similar aggregation methods given the underlying dynamics of compartmental transmission models (Weissman et al., 2020; Chinazzi et al., 2020; Salje et al., 2020). We have observed that models in which acquisition of immunity has a substantial impact on transmission dynamics or in which interventions are triggered when a threshold burden of infection is reached are particularly prone to producing asynchronous trajectories that are difficult to summarise in ways that do not obscure relevant properties of individual trajectories. These effects are often amplified when using stochastic models (Fig. 1).

Alternative means of communicating uncertainty have been explored throughout the COVID-19 pandemic. For example Juul et al. (2021), use central ranking systems which consider entire epidemic curves in relation to one another to produce curve boxplots and compare the likelihood of individual simulations in relation to key metrics of interest (i.e. daily number of new cases requiring hospitalisations). On the other hand Davies et al. (2020), plot a subset of ‘representative’ curves drawn from each decile corresponding to the total number of simulated new cases. Additionally Koo et al. (2020), show each realisation of their simulations and use darker colours to indicate those which are closer to the median. While these approaches enable variation in epidemic trajectories to be communicated, we believe that sometimes the interpretability of the results to non-technical audiences is hindered. We argue that focussing directly on the metrics that inform policy decisions and making judicious choices of what quantities are plotted allows results, both in terms of central estimates and uncertainty to be communicated accurately, whilst ensuring these results are accessible to non-technical audiences.

In Fig. 3, we instead present the individual trajectories of ICU demand over the simulation period alongside individual metrics of magnitudes and peak timings. In an effort to engage the audience more with the uncertainty, the colour of each epidemic trajectory reflects the ranked magnitude of the first peak allowing the key metrics presented in Figs. 3c and 3d to be more easily linked to the trajectory’s epidemic dynamics in Figs. 3a and 3b. Fig. 3 paints a different picture of the potential unfolding epidemics than that provided via the simple summary statistics in Fig. 2. This is particularly applicable to the “reactive” strategy, which observes a high degree of variability across trajectories which is not clearly conveyed using summary statistics calculated at each time point. The individual metrics of magnitude and peak timing allow for a clearer interpretation of the necessary support that national-level decision-makers ought to allow hospital-level decision-makers to prepare for a surge in demand, depending on the NPI strategy they implement. For example, the “scheduled” strategy suggests public health action should focus on providing additional hospital beds (Fig. 3c) whereas the “reactive” strategy suggests that officials may also need to consider how quickly they need to procure additional hospital beds (Fig. 3d).

**Fig. 4:**
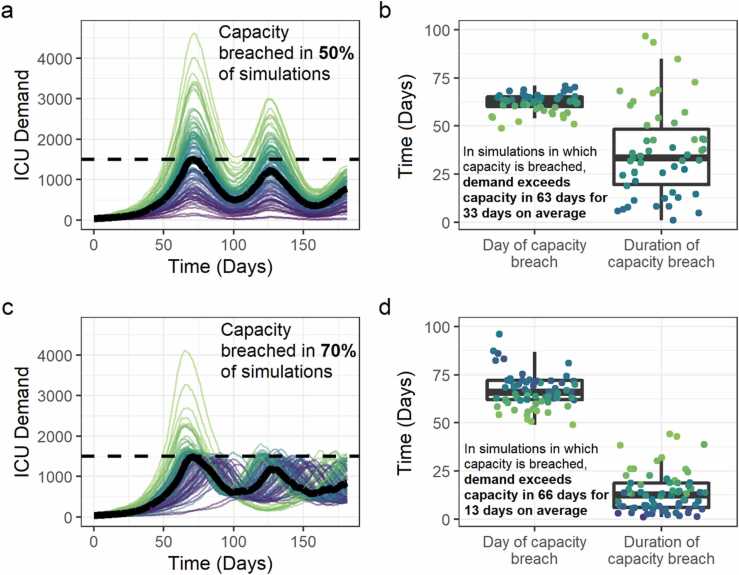
Linking epidemic projections of ICU demand under two suppression strategies to potential capacity breaches. Individual simulations of intensive care unit (ICU) demand from COVID-19 patients compared to an assumed ICU capacity of 1500 available beds is shown for strategies in which suppression measures are (a) scheduled at a fixed time point and (c) triggered reactively. In simulations in which capacity is breached, corresponding metrics of the day on which capacity is breached and the duration of the capacity breach shown in (b) and (d), respectively. In (a) and (c) the median trajectory is shown with a black line. The colour of each simulation realisation depicts the ranking of ICU demand at first peak [high in green to low in purple] and allows for the metrics in (b) and (d) to be more easily linked to the trajectories in (a) and (c) respectively.

To further aid public health officials, in Fig. 4 we present our simulations in conjunction with measures of ICU capacity. Under the assumption of 1500 available beds, 50% of the simulations from the “scheduled” strategy estimate that capacity will be breached in comparison to 70% of simulations from “reactive” strategy (Figs. 4a and 4c). In Figs. 4b and 4d, the first day and duration of capacity breaches are presented individually for those simulations in which capacity is exceeded, again using colour to link to the individual trajectories as in Fig. 3. In each plot, we have used text to provide brief, key messages to the audience without obscuring the full range of uncertainty underlying each message. Together, this provides a more detailed insight into the range of scenarios that ICUs may be faced with than that which could be gained from solely looking at the individual trajectories and may be particularly useful to national-level decision-makers in deciding which NPI strategy to implement. Similar to the timing of the first peak (Figs. 3c and 3d), there is greater variability in the first day of a possible capacity breach under the “reactive” strategy compared to the “scheduled” strategy. However, although more simulations breach capacity under the “reactive” strategy, the duration of the breach is anticipated to be shorter compared to the “scheduled” strategy given the model assumptions. Supplementary Figures 2 and 3 are analogous to Fig. 3, Fig. 4 but instead consider general hospital demand.

We believe these simple visualisations allow for the key metrics to be more easily understood while being transparent about the uncertainty in our epidemic trajectories. Although we have chosen in this instance to focus on the dynamics of ICU demand at the first peak, understanding the audience is still paramount and it is imperative that visualisations are tailored as such. For example, decision-makers may be more interested in the outcomes of specific interventions and contrasting alternative scenarios. This is where interactive tools become increasingly valuable and allow users to explore scenarios specific to their needs, such as surrounding COVID-19 vaccine allocation strategies COVID-19 Scenario Analysis Tool. However, greater flexibility introduces further complexities in striking the correct balance between informing and not overwhelming audiences in chosen data visualisations (Fox and Hendler, 2011).

Although the clear communication of uncertainty was important long before the COVID-19 pandemic, the severity and rapidly-evolving nature of this emergency has underlined its importance. Brooks-Pollock et al. (2021) note that “communication to non-specialists became an overnight skill required of disease modellers”. Some scientific advisors in the UK have noted the improvement in both advisor’s and decision-maker’s understanding of mathematical modelling over the course of the pandemic, but as noted by other former advisors and current members of the UK Scientific Advisory Group for Emergencies (SAGE), the communication of uncertainty inherent in these models requires improvement (McCabe and Donnelly, 2021). The responsibility of communication has traditionally fallen solely on scientists, but it has been noted that developing the general scientific understanding of decision-makers could also ease the intense pressure placed upon the scientific advisors when responding to an emergency (McCabe and Donnelly, 2021). Additionally, it may be more appropriate to train intermediaries for specialist science communication and data visualisation roles in order to share the substantial burden that such activities place on both scientists’ and decision-makers’ time.

As the pandemic continues, mathematical models of transmission to inform policy tend to become increasingly complex to account for vaccination and the emergence of variants of SARS-CoV-2 with different phenotypes while still modelling the impact of population behaviour, for example through the implementation of non-pharmaceutical interventions. Clear communication of uncertainty will remain important now and also in future health emergencies. We hope here to have added to a much-needed discussion about effective visualisation approaches for conveying uncertainty.

## CRediT authorship contribution statement

**Ruth McCabe:** Formal analysis, Writing – original draft, Writing – review & editing. **Mara D. Kont:** Writing – review & editing. **Nora Schmit:** Writing – review & editing. **Charles Whittaker:** Writing – review & editing. **Alessandra Løchen:** Writing – review & editing. **Patrick G.T. Walker:** Writing – review & editing. **Azra C. Ghani:** Writing – review & editing. **Neil M. Ferguson:** Writing – review & editing. **Peter J. White:** Writing – review & editing. **Christl A. Donnelly:** Writing – review & editing. **Oliver J. Watson:** Conceptualization, Formal analysis, Writing – original draft, Writing – review & editing.

## Declaration of Competing Interest

The authors declare no competing interests.

## Data Availability

Model fitting and analysis is performed in R. Data and code to reproduce figures are available at https://github.com/j-idea/europe-icu-capacity.
