# Supplementary material for: Communicating uncertainty in epidemic models
Source: Epidemics. 2021 Dec;37:100520. doi: 10.1016/j.epidem.2021.100520 (PMC8562068; doi:10.1016/j.epidem.2021.100520)
Supplement: Supplementary file 1 — Supplementary material [file mmc1.docx]

**Communicating uncertainty in epidemic models**

**Supplementary Material**

Ruth McCabe^*^, Mara D. Kont, Nora Schmit, Charles Whittaker, Alessandra Løchen, Patrick G. T. Walker, Azra C. Ghani, Neil M. Ferguson, Peter J. White, Christl A. Donnelly, Oliver J. Watson

*Corresponding author: Ms. Ruth McCabe, Department of Statistics, University of Oxford, 24-29 St Giles', Oxford, OX1 3LB, UK. E-mail: [ruth.mccabe@linacre.ox.ac.uk](mailto:ruth.mccabe@linacre.ox.ac.uk)


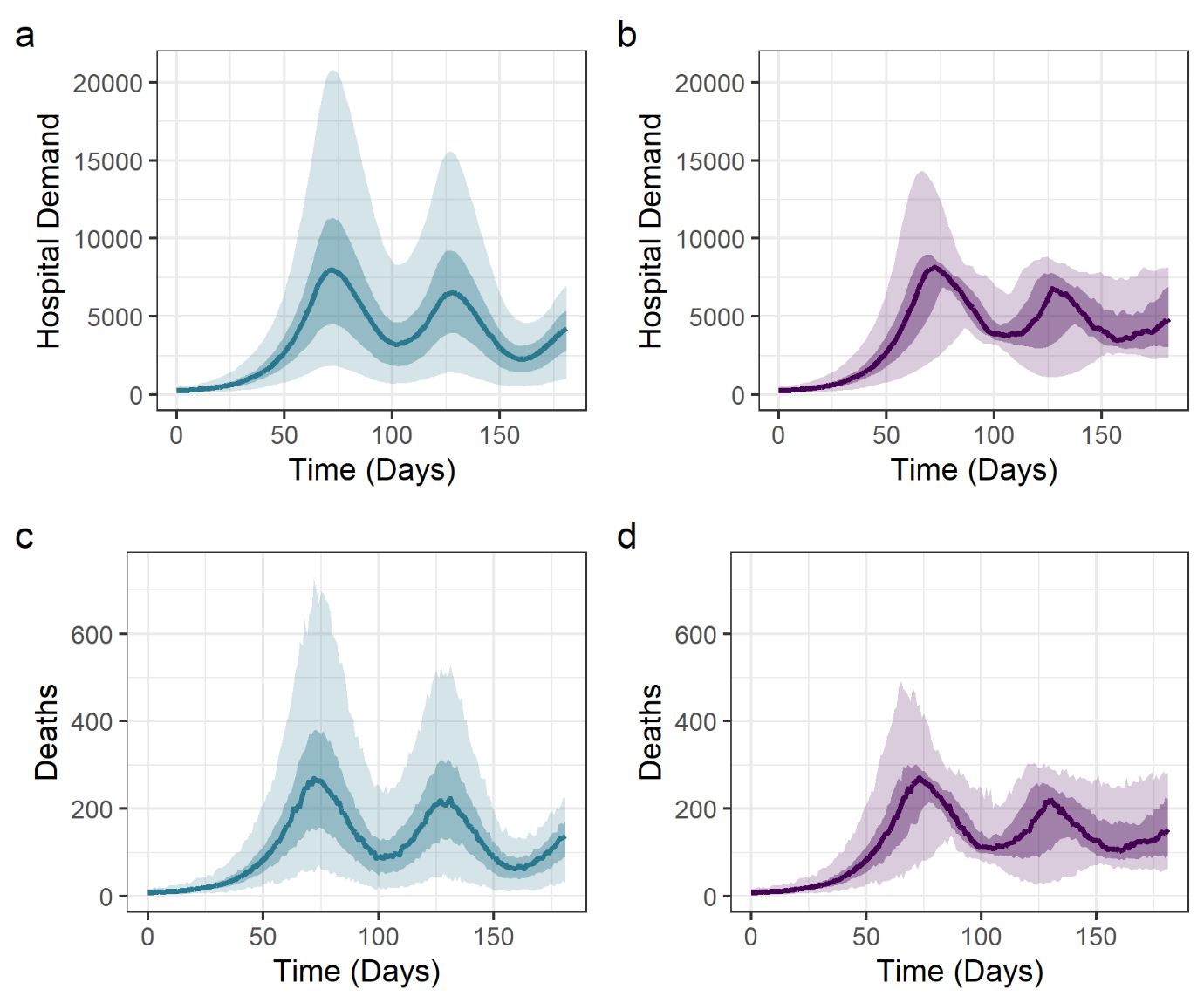


**Supplementary Figure 1: Traditional presentation of epidemic forecasts under two suppression strategies.** The median (line) with 50% credible intervals (dark shading) and 95% credible intervals (light shading) of 100 simulations of hospital demand from COVID-19 patients and COVID-19 deaths are shown for strategies in which suppression measures are (a) and (c) scheduled at a fixed time point and (b) and (d) triggered reactively.


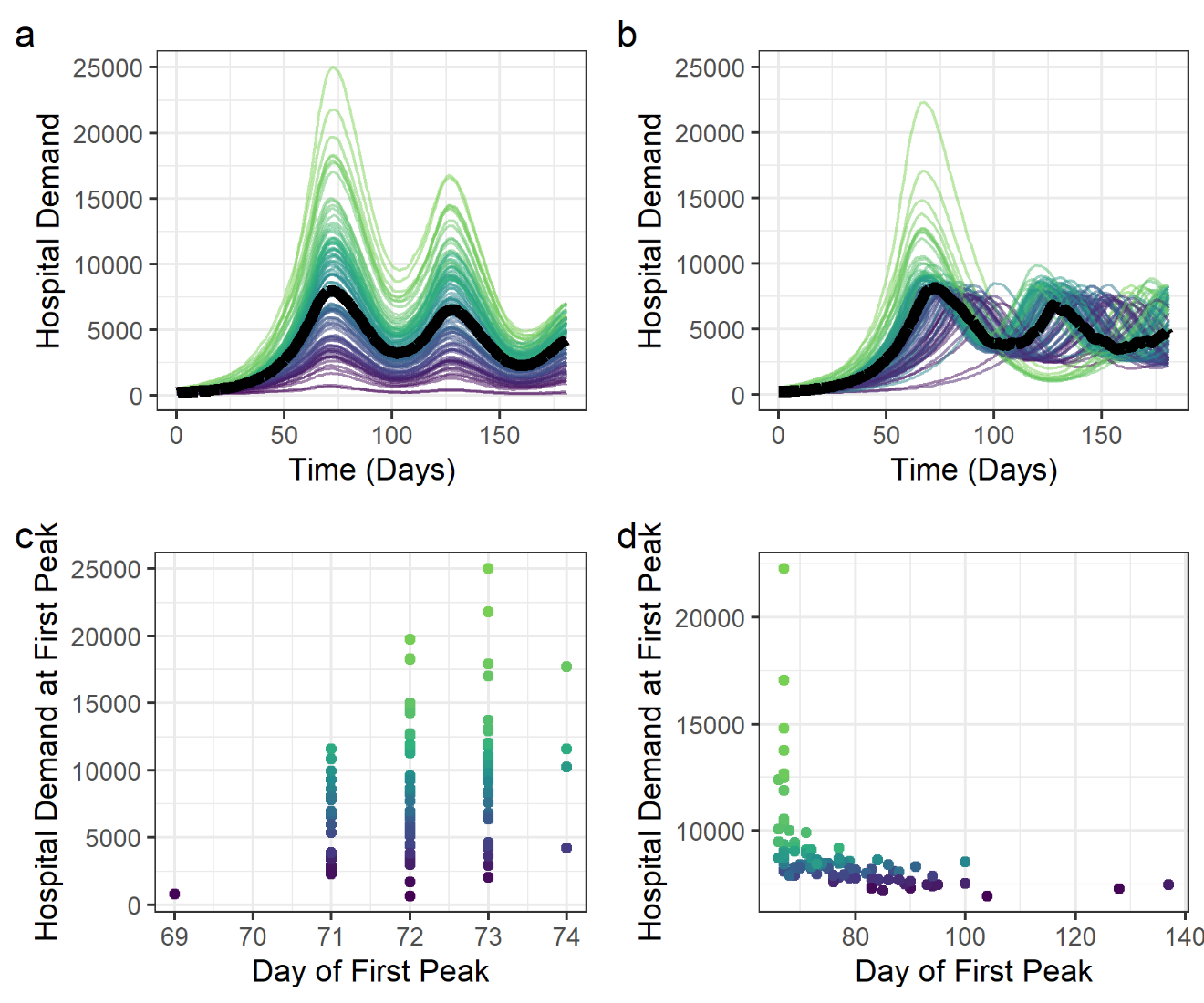


**Supplementary Figure 2: Communicating uncertainty in epidemic forecasts of hospital demand under two suppression strategies.** Individual simulations of hospital demand from COVID-19 patients is shown for strategies in which suppression measures are (a) scheduled at a fixed time point and (b) triggered reactively, with corresponding relationships between the magnitude and timing of peak hospital demand for each realisation shown in (c) and (d), respectively. In (a) and (b) the median trajectory is shown with a black line. The colour of each simulation realisation depicts the ranking of hospital demand at first peak [high in green to low in purple] and allows for the metrics in (c) and (d) to be more easily linked to the trajectories in (a) and (b) respectively.


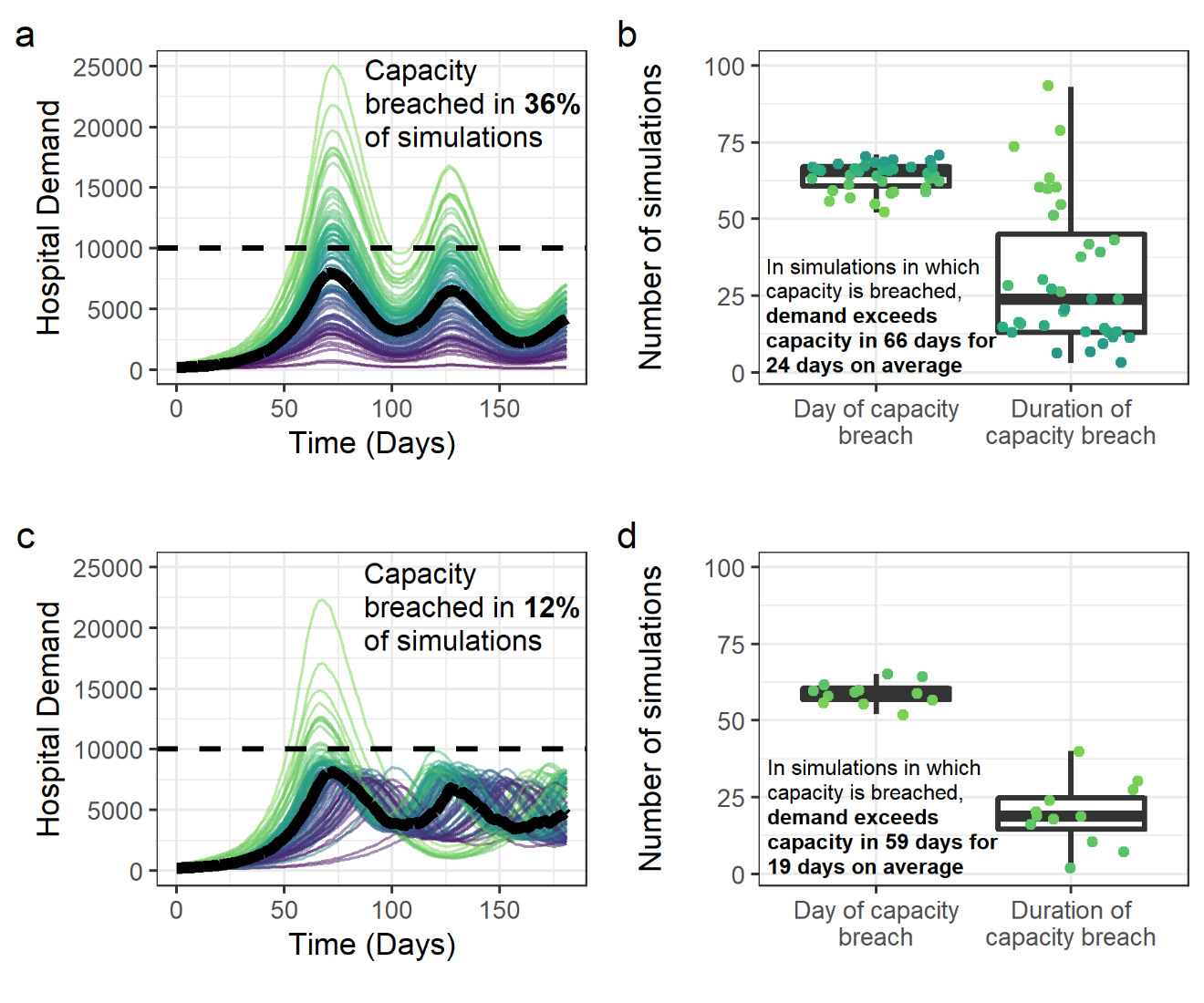


**Supplementary Figure 3: Linking epidemic forecasts of hospital demand under two suppression strategies to potential capacity breaches.** Individual simulations of hospital demand from COVID-19 patients compared to an assumed hospital capacity of 10000 available beds is shown for strategies in which suppression measures are (a) scheduled at a fixed time point and (c) triggered reactively. In simulations in which capacity is breached, corresponding metrics of the day on which capacity is breached and the duration of the capacity breach shown in (b) and (d), respectively. In (a) and (c) the median trajectory is shown with a black line. The colour of each simulation realisation depicts the ranking of hospital demand at first peak [high in green to low in purple] and allows for the metrics in (b) and (d) to be more easily linked to the trajectories in (a) and (c) respectively.
